# Effect of Dapagliflozin on Type 2 Diabetes Mellitus With Nonalcoholic Fatty Liver Disease: A Single-Center Survey

**DOI:** 10.7759/cureus.14974

**Published:** 2021-05-11

**Authors:** Chandan Das, Debasmita Tripathy, Surendranath Swain, Navin Sudhakaran, Kanishka Uthansingh, Pradeep Mallick, Girish K Pati

**Affiliations:** 1 Department of Medicine, Institute of Medical Sciences and SUM Hospital, Siksha 'O' Anusandhan Deemed to be University, Bhubaneswar, IND; 2 Department of Gastroenterology and Hepatobiliary Sciences, Institute of Medical Sciences and SUM Hospital, Siksha 'O' Anusandhan Deemed to be University, Bhubaneswar, IND; 3 Department of Gastroenterology and Hepatobiliary Sciences, Institute of Medical Sciences and SUM Hospital, Bhubaneshwar, IND

**Keywords:** dapagliflozin, fibroscan, hepatic fibrosis, steatosis

## Abstract

Introduction

Nonalcoholic fatty liver disease (NAFLD) is the most common chronic liver disease, with a global prevalence of 20%-40%. Approximately 40%-60% of patients with type 2 diabetes mellitus (DM2) experience NAFLD; out of which 20%-40% cases may have higher severity. Due to the scarcity of available reports from the eastern part of India, we aimed to evaluate the effects of dapagliflozin, a sodium-glucose cotransporter-2 inhibitor used in these types of cases.

Material and methods

The study included consecutive patients with DM2 and NAFLD, treated with dapagliflozin at 10 mg daily for six months. All patients underwent detailed anthropometric, biochemical, abdominal ultrasonography, and transient elastography studies at baseline and after therapy as well as a comparative analysis.

Results

In the 100 patients included in our study, the male patients outnumbered the female patients (male-to-female ratio, 4.27:-1) and the mean age at presentation was 44.11 ± 8.24 years. The mean body mass index significantly decreased over the course of the therapy, from 27.31± 1.87 kg/m^2^ at baseline to 26.21 ± 1.51 kg/m^2^ after the therapy. The patients’ transaminitis, dyslipidemia, and glycemic status significantly improved over the course of the therapy. We also observed significant (p < 0.05) improvement in hepatic steatosis by the end of the treatment. Although transient elastography by FibroScan-measured hepatic fibrosis score (Echosens, Paris, France) significantly decreased from 6.95 ± 1.42 to 6 ± 1.44 kPa, hepatic fibrosis did not improve significantly (p ≥ 0.05) following therapy.

Conclusion

Although dapagliflozin improved body mass index, transaminitis, dyslipidemia, glycemic status, and hepatic steatosis, it had a minimal effect on hepatic fibrosis.

## Introduction

Nonalcoholic fatty liver disease (NAFLD) is currently the most prevalent chronic liver disease, with a global burden of 17 % to 46% of chronic liver disease cases [1]. Although NAFLD can be asymptomatic and remain undetected during the initial stages of the disease, approximately 20% to 30% of patients with NAFLD can experience nonalcoholic steatohepatitis, which can progress to significant hepatic fibrosis or cirrhosis and occasionally to hepatocellular carcinoma in the long term if the disease remains unaddressed [2,3]. Patients with type 2 diabetes mellitus (DM2) have a very high prevalence of NAFLD, which can vary from 40% to 60% [4].

As NAFLD typically has a very long asymptomatic course and may silently lead to hepatic steatohepatitis and fibrosis, early diagnosis by noninvasive methods and implementation of appropriate therapy is the need of the hour [5]. Although numerous drugs have been assayed to treat NAFLD, none has been unanimously approved for treating the disease with reasonable efficacy and safety. Finding a suitable efficacious and safe drug to treat NAFLD is, therefore, the need of the hour. Sodium-glucose cotransport-2 (SGLT2) inhibitors are a specific type of oral ant diabetic drug, which can improve the glycemic status due to the drugs’ significant glycosuric effect [6]. Published reports have suggested that SGLT2 inhibitors might prevent the development of NAFLD in rats and even improve the histological activity in obese mice with type 2 DM2 [7, 8]. Although two previous trials in human patients with DM2 and NAFLD showed positive effects for SGLT2 inhibitors on hepatic steatosis, they did not assay the drugs’ antifibrotic effects [9, 10].

Given that the early diagnosis of NAFLD is an essential precautionary measure, the disease should be thoroughly scrutinized in susceptible patients, with available conventional diagnostic modalities such as ultrasonography elastography, and noninvasive fibrosis markers. Transient elastography by FibroScan typically evaluates hepatic steatosis by quantifying the controlled attenuation parameter (CAP) and hepatic fibrosis by estimating liver stiffness (LS), which can accurately predict the thepatic fibrosis stage [11]. An invasive liver biopsy study, which is considered the gold standard for assessing NAFLD, might not be required if transient elastography is meticulously performed, given the significant correlation between the measurements and NAFLD [11]. Therefore, CAP and LS measurement by transient elastography may be a safer option in the evaluation of hepatic steatosis and fibrosis respectively in patients with DM2, who are more prone to suffer from a higher degree of hepatic steatosis and fibrosis [12, 13]. Recently, several noninvasive biochemical scoring methods have been made available for detecting hepatic fibrosis, such as the NAFLD fibrosis score and the fibrosis-4 (FIB -4) index [14]. Currently, the available non-invasive methods for assessment of hepatic steatosis and fibrosis are much easier and convenient compared to the gold-standard liver biopsy study in NAFLD cases.

 In view of the paucity of data on the role of dapagliflozin in cases of DM2 with NAFLD in the eastern part of India, we aimed to analyze the variable effects of dapagliflozin in this specific population group.

## Materials and methods

This prospective, single-center, open-label, uncontrolled, interventional, multidisciplinary cohort study was conducted in the Department of ‘General Medicine’ and ‘Gastroenterology and Hepatobiliary Sciences’ of the Institute of Medical Sciences and SUM Hospital, Bhubaneswar, Odisha, India. The study included and prospectively evaluated consecutive patients with DM2 and NAFLD (who attended the outdoor clinic) of the above-mentioned department between January 2020 and June 2020.

By subjecting the patients to appropriate investigations, NAFLD was diagnosed on the basis of ultrasound evidence of hepatic steatosis, after ruling out other causative factors of hepatic steatosis, such as significant alcohol consumption (daily intake >-20g for men and >-10g for women), negative hepatitis B and C viral serology, autoimmune hepatitis, hepatic storage disorders, such as hemochromatosis and Wilson’s disease, the consumption of suspicious drugs, and other diseases that might be associated with hepatic steatosis. DM2 was diagnosed based on fasting blood glucose level ≥126 mg/dL and postprandial or 2-h postprandial blood glucose levels and random blood glucose levels of 200 mg/dL on at least two occasions [4]. The patients younger than 18 years or older than 65 years, who are on medications that could potentially affect NAFLD such as vitamin E, metformin, pioglitazone, saroglitazar, ursodeoxycholic acid, obeticholic acid, and glucagon-like peptide 1 analogues were excluded from the study. All patients underwent detailed biochemical and radiological tests following 12 hours of fasting once at baseline and again after six months of therapy with dapagliflozin at 10 mg per day.

We calculated the noninvasive fibrosis markers for NAFLD according to the following formulas. For the NAFLD fibrosis score = the equation was -1.675 + 0.037 × age(years) +0.094 × body mass index (BMI (kg/m2) + 1.13 × impaired fasting glucose/diabetes (yes = 1, no = 0) + 0.99 × aspartate aminotransferase (AST)/alanine transaminase (ALT) ratio -0.013 × platelet count (109/ L) -0.66 × albumin (g/dL) For the FIB- 4 index, the equation was (age {years} × AST {units/L}/platelet count (×109/L) × (ALT {units/L}1/2. We also assayed the aspartate aminotransferase to platelet ratio index (APRI) to quantify the hepatic fibrosis in our study. Hepatic steatosis was graded into three levels as per abdominal ultrasound findings as follows: (1) mild, minimally increased hepatic parenchymal echogenicity with a normal-looking diaphragm and portal vein wall; (2) moderate, moderately increased hepatic echogenicity with a minimally-blurred portal vein wall and the diaphragm; and (3) severe, markedly increased hepatic echogenicity with a very poorly visualized portal vein wall, diaphragm, and posterior part of the right liver lobe [15]. Transient elastography was carried out with FibroScan ultrasound machine with a standard 3.5-MHz M probe (Echosens, Paris, France) to assess CAP and LS simultaneously. The hepatic steatosis was graded into the following three standard types according to the FibroScan CAP assay as follows: (1) mild; (2) moderate or significant; and (3) severe, using the optimal cut-off values of 248 (237-261) dB/m (S > 0), 268 (257-284) dB/m (S > 1), and 280 (268-294) dB/m (S > 2), respectively [16]. Written informed consent was obtained from all the study participants prior to inclusion in the study. Necessary approval was obtained from the Institutional Ethics Committee, IMS and SUM Hospital, Bhubaneswar, prior to enrolling the participants in the study.

 Statistical analysis

The statistical analysis was performed using SPSS software version 23 (IBM Corp., Armonk, NY). We calculated the sample size to be 71 for this interventional study on the basis of NAFLD prevalence in the community; which was around 25-30% and expected post-therapy drop in ALT level by 10% for the power of the study to be 80% and alpha to be 0.05. The results are presented as mean ± standard deviation or frequency in percentages. We compared the normally distributed quantitative and categorical variables at baseline and after therapy, using paired Student’s t-test and chi-square test, respectively. P values<-0.05 were considered significant in our study.

## Results

One hundred consecutive patients with DM2 and fatty liver were evaluated prospectively in this study. Males outnumbered the females (male-to-female ratio: 4.27:1) in our study. Mean age of the study population at baseline was 44.11 ± 8.24 years. Mean BMI decreased from 27.31 ± 1.87 kg/m2 to 26.21 ± 1.51 kg/m2 (p=0.0001) following six months of dapagliflozin therapy. The mean total platelet count (TPC) and serum bilirubin at baseline were 200,100±54,810/µL and 1.24 ± 0.34 mg/dL respectively. The mean serum liver enzymes at baseline in IU/L were as follows: SGOT - 55.88 ± 20.92, SGPT - 64.69 ± 22.73 and GGT - 67.54 ± 17.72. Mean high-density lipoprotein cholesterol (HDL-C) and triglyceride (TG) level at baseline were 43.52 ± 10.19 mg/dL, and 172 ± 21.41 mg/dL respectively. The mean serum albumin level at baseline was 3.72 ± 0.46 g/L. Mean FibroScan CAP score at baseline and after six months of therapy was 273.11 ± 17.51 dB/m and 251.16 ± 11.7 dB/m, respectively (p = 0.0001). Table 1 lists the biochemical parameters at baseline and after six months of dapagliflozin therapy. Table 2 describes the abdominal ultrasound findings at baseline and after six months of dapagliflozin therapy. Table 3 describes hepatic steatosis grade as per the FibroScan controlled attenuation parameter assay at baseline and after dapagliflozin therapy. Table 4 describes baseline FibroScan score and noninvasive scoring for fibrosis in all the cases at the time of evaluation and following dapagliflozin therapy. Table 5 describes stages of hepatic fibrosis by various assays at baseline and after dapagliflozin therapy. Hepatic fibrotic stages ‘I-II’ and ‘III-IV’ were defined as “non-significant and “significant” hepatic fibrosis respectively in our study.

**Table 1:** Biochemical parameters at baseline and after six months of dapagliflozin therapy SGOT: serum glutamate oxaloacetate transaminase; IU/L: international unit/ litre; SGPT: serum glutamate pyruvate transaminase; GGT: gamma glutamyl transpeptidase; FBS: fasting blood sugar; MG/DL: miligram/decilitre; PPBG: postprandial blood glucose; HDL: high density lipoprotein cholesterol; TG: triglyceride

Biochemical parameters	At baseline	After six months of therapy	p
SGOT (IU/L)	55.88 ± 20.92	49.52 ± 16.61	0.0001
SGPT (IU/L)	64.69 ± 22.73	58.69 ± 17.98	0.0001
GGT (IU/L)	67.54 ± 17.72	43.21 ± 12.49	0.0001
FBG (mg/dL)	137 ± 18.91	125.54 ± 15.87	0.0001
2 hour PPBG (mg/dL)	188.38 ± 34.49	169.13 ± 29.49	0.0001
Serum HDL (mg/ dL)	43.52 ± 10.19	47.73 ± 7.6	0.0001
Serum TG (mg/dL)	172 ± 21.41	147.5 ± 22	0.0001

**Table 2:** Hepatic steatosis grade as per abdominal ultrasonographic findings at baseline and following six months of dapagliflozin therapy

Grade of fatty liver	At baseline (% cases)	After six months of therapy (% cases)	p-value
Mild	44	82	<0.0001
Moderate	41	17	0.0002
Severe	15	01	0.0003

**Table 3:** Hepatic steatosis grade as per the Fibroscan-controlled attenuation parameter assay (CAP) at baseline and following dapagliflozin therapy

Grade of fatty liver	At baseline (% cases)	After six months of therapy (% cases)	p-value
Mild (S_1_)	42	82	<0.0001
Moderate (S_2_)	43	17	<0.0001
Severe (S_3_)	15	01	0.0003

**Table 4:** Baseline Fibroscan score and non-invasive fibrosis scoring at baseline, following dapagliflozin therapy kPa: kilopascals; APRI: aspartate aminotransferase to platelet ratio index; NAFLD: nonalcoholic fatty liver disease

Fibrosis scores	At baseline	After six months of therapy	p-value
FibroScan values (kPa)	6.95 ± 1.42	6 ± 1.44	0.001
APRI Score	0.79 ± 0.52	0.7 ± 0.41	0.15
FIB4 Score	1.66 ± 0.91	1.54 ± 0.76	0.29
NAFLD Fibrosis Score	0.58 ± 0.92	0.64 ± 0.91	0.63

**Table 5:** Stages of hepatic fibrosis by various assays at baseline and following dapagliflozin therapy NAFLD: nonalcoholic fatty liver disease; APRI: aspartate aminotransferase to platelet ratio index

Stages of hepatic fibrosis	At baseline (% cases)	Post-therapy (% cases)	p-value
Non significant fibrosis as determined by FibroScan Assay	84	90	0.2
Significant fibrosis as determined by FibroScan Assay	16	10	0.2
Non significant fibrosis as determined by FIB4 assay	89	92	0.46
Significant fibrosis as determined by FIB4 assay	11	8	0.46
Non significant fibrosis as determined by NAFLD fibrosis assay	83	86	0.55
Significant fibrosis as determined by NAFLD fibrosis assay	17	14	0.55
Indeterminate or no hepatic fibrosis as determined by APRI assay	51	67	0.02
Significant hepatic fibrosis as determined by APRI assay	49	33	0.02

## Discussion

This study is first of its kind to comprehensively evaluate the effects of the SGLT2 inhibitor dapagliflozin on both hepatic steatosis and fibrosis using ultrasonography and transient elastography study and by analyzing the results of various noninvasive hepatic fibrosis scales in patients with DM2 and NAFLD. Most of the patients in our study were male and around 40 to 50 years old. We found significant improvement in various liver enzyme biochemical parameters after dapagliflozin therapy, as similarly reported in previous studies [17-21]. Even in patients with NAFLD but no diabetes, significant liver enzyme improvement following dapagliflozin therapy has been observed [17]. The authors concluded that the beneficial hepatic effects of dapagliflozin might be unrelated to the patient’s glycemic and anthropometric status [17]. Patients’ glycemic and anthropometric status (such as BMI) following dapagliflozin therapy were significantly improved in our study as similarly reported by Tobita et al. and Sattar et al. [18, 20]. Most of our study patients had mild to moderate hepatic steatosis, as reported by the abdominal ultrasounds and FibroScan CAP assays, which showed significant improvement following dapagliflozin therapy. Prior studies with SGLT2 inhibitors in patients with DM2 and NAFLD have also shown significant improvement in hepatic steatosis, which further support our findings [10, 22]. Although we observed significant improvement in the FibroScan-assisted hepatic fibrosis scores, the patients’ hepatic fibrosis did not improve significantly following the dapagliflozin therapy. Hepatic fibrosis was not improved significantly following dapagliflozin therapy when measured by a different non-invasive scoring method, except the APRI scoring method. Although a non-significant reduction in the APRI score was observed following dapagliflozin therapy hepatic fibrosis was significantly improved following dapagliflozin therapy when assayed by the APRI scoring method. We observed a significant reduction in the FibroScan-measured hepatic fibrosis score, as did a study by Shimizu et al., whose results strongly support our findings [22]. The study by Wang et al. also supported our results, reporting a decrease in aminotransferase level, hepatic steatosis, and fibrosis in obese rodents, following dapagliflozin therapy, similar to the findings in our study in humans with DM2 and NAFLD [23]. Our study findings also agreed with those of Tobita et al., who observed significant improvement in liver enzyme levels, obesity, and glycemic status after six months of dapagliflozin therapy in patients with DM2 with biopsy-proven nonalcoholic steatohepatitis [18]. However, we were unable to evaluate the hepatic status through a liver biopsy study in our study population, which was one of the major limitations in our study. We also observed a significant reduction in serum TG levels and an increase in serum HDL-C levels, following dapagliflozin therapy, similar to those reported in the study by Gameil et al., results that might be related to the improved glycemic status and weight loss following dapagliflozin therapy [17]. In our study, we noticed significant body weight reduction, following SGLT2 inhibitor therapy similar to that noticed by Ferrannini et al., findings that might be related to the significant decrease in total body fat mass due to the greater use of fats than carbohydrates [24]. Previous reports have suggested an improvement in hepatic fibrosis, both histologically [25] and by available surrogate markers analysis [22], although the exact pathophysiologic mechanism by which SGLT2 inhibitors improve hepatic steatosis and fibrosis has not been fully clarified. In this study, we described the various suggested pathophysiologic mechanisms of the actions of SGLT2 inhibitors in NAFLD, Which might help in the up-regulation of effective fatty acid oxidation over carbohydrate oxidation, thereby leading to reduced hepatic steatosis and inflammation [26]. These SGLT2 inhibitors can not only effectively reduce glucose oxidation and oxidative stress, but also accelerate lipolysis and effective free fatty acid oxidation, therapy resulting in a marked improvement in NAFLD [24]. These inhibitors might effectively reduce visceral adiposity, insulin resistance, and body weight due to their prominent glycosuric effect, which promotes beta-oxidation in the liver and improves NAFLD [27]. The SGLT2 inhibitors’ glycosuric effect can also improve hepatic steatosis by reducing fatty acid production. In our study, although dapagliflozin therapy resulted in marked improvement in hepatic steatosis, but failed to show significant improvement in hepatic fibrosis, as the hepatic fibrosis process is usually an irreversible process, which may not be modified significantly following any medication. Although marked improvement in anthropometric, metabolic, and biochemical parameters took place following dapagliflozin therapy in our study, hepatic fibrosis was not altered much following therapy. This might be related to the shorter treatment duration or lower dosage and certain undiscovered confounding elements in the etiopathogenesis of NAFLD, which cannot be convincingly addressed by dapagliflozin therapy. These issues ought to be explored in future long-term prospective studies for a better treatment plan of this benign-looking fatal disorder.

Limitations

This study has some limitations, which were as follows: open-label, non-randomized and uncontrolled study design; lack of long-term therapy and post-therapy follow-up and nonperformance of liver biopsy for histological confirmation, which is considered as the gold standard for NAFLD assessment. However, robust evidence suggested that transient elastography by FibroScan study is equally efficacious as liver biopsy study to assess liver fibrosis [11]. We also did not assess the effects of dapagliflozin on nondiabetic patients with NAFLD, given that these patients were excluded from the study.

Strength of the study

Possibly, this study is the first of its kind to vividly and comprehensively assess various noninvasive fibrosis biomarkers for NAFLD, along with a comparative assessment of the various biochemical parameters, anthropometric variables, abdominal ultrasonography, and simultaneous FibroScan study, both at baseline and after completing the therapy.

## Conclusions

Although this study was a short-term, non-randomized, interventional uncontrolled study, we could observe significant improvement in hepatic steatosis following dapagliflozin therapy, as reported by both ultrasonography and FibroScan assessment. Dapagliflozin is possibly non-antifibrotic in nature, as we failed to observe significant improvement in hepatic fibrosis with its use in our study. However, our study findings should be validated in the future by long-term, randomized, well-controlled interventional trials for better judgment of its use in the diabetic NAFLD population. 
